# The crucial role of age and site in incidence and prognosis of female neuroendocrine neoplasms in the United States: a population-based study from 2000 to 2018

**DOI:** 10.18632/aging.205573

**Published:** 2024-03-01

**Authors:** Guixiu Xiao, Zihan Xu, Yong Zhang, Shuang Dai, Ganlu Ouyang, Yan Huang, Yanyang Liu, Dan Cao, Feng Luo

**Affiliations:** 1 Department of Medical Oncology, Cancer Center, West China Hospital, Sichuan University, Chengdu, Sichuan, China; 2 Lung Cancer Center, West China Hospital, Sichuan University, Chengdu, Sichuan, China; 3 Laboratory of Integrative Medicine, Clinical Research Center for Breast, State Key Laboratory of Biotherapy, West China Hospital, Sichuan University and Collaborative Innovation Center, Chengdu, Sichuan, China; 4 Abdominal Oncology Ward, Division of Medical Oncology, Cancer Center, State Key Laboratory of Biotherapy, West China Hospital, Sichuan University, Chengdu, Sichuan, China

**Keywords:** neuroendocrine neoplasms (NENs), morbidity trends, prognosis, age specificity, nomograms

## Abstract

Background: As the incidence continues to rise, global concern about neuroendocrine neoplasms (NENs) is mounting. However, little is known about how NENs affect women patients.

Methods: The annual percentage change (APC) was calculated to describe the incidence. Cox proportional hazards multivariable regression was used to identify risk factors. The nomograms were employed to estimate prognosis.

Results: A total of 39,237 female NENs (fNENs) cases were identified. The incidence of fNENs increased annually (APC = 4.5, 95% CI 4.1-4.8, P < 0.05), and the incidence pattern and survival outcomes showed age and site-specificity. Appendiceal, rectal, and pulmonary fNENs were major contributors to the incidence of patients younger than 40, between 40-59, and over 60 years old, respectively. The Cox proportional hazards regression model revealed that age, tumor size, grade, stage, and primary sites were closely related to survival. The worst survival outcomes appeared in breast, reproductive system, and liver fNENs for patients under 40, between 40-49, and over 50 years old, respectively. A nomogram based on these developed with higher predictive accuracy of prognosis, with a C index of 0.906 in the training cohort and 0.901 in the validation cohort.

Conclusions: Our findings revealed distinct site-specific tendencies in the incidence and survival patterns among fNEN patients across various age groups. Thus, reasonable patient screening and stratification strategies should be implemented, especially for young patients.

## INTRODUCTION

Neuroendocrine neoplasms (NENs) are rare cancers derived from neuroendocrine cells that share features of neural and endocrine differentiation [1]. NENs consist of a heterogeneous group of neoplasms that can occur in almost all sites, most frequently in the digestive and respiratory system [2, 3]. The steady increase of NENs over the past decades has received global attention, accordingly, tremendous efforts have been made in superior classification, molecular diagnosis, radionuclide, and targeted therapies of NENs [3–7]. However, the epidemiological characteristics and prognostic factors, crucial for early perception and risk stratification, have not been fully understood, particularly for women patients.

Gender heterogeneity exists in the incidence, prevalence, and outcomes of various types of cancer, including NENs [8–11]. For instance, the increased incidence of women patients was almost twice as much as men in lung atypical carcinoid and typical carcinoid [12]. Another study showed that the incidences between genders were almost similar previously, but the incidence in females has increased at a more rapid rate than in males since 2013 [13]. Understanding these differences can aid the development of more effective prevention and treatment strategies for both male and female patients. Unfortunately, previous studies had enrolled both men and women, but only as a covariate instead of conducting specifically gender-specific analysis [14–16]. Therefore, there is a critical need to investigate the epidemiological characteristics of female NENs (fNENs) for more accurate clinical recognition and treatment.

Here, we performed a population-based study using the data from the Surveillance, Epidemiology, and End Results (SEER) database to analyze the clinicopathologic features and prognostic characteristics of fNENs. Our goal was to provide a comprehensive description of demographic characteristics and recent incidence trends of fNENs and establish a nomogram model for prognostic assessment to assist clinical decision-making.

## MATERIALS AND METHODS

### Data source

The SEER database, a national cancer registry, is an authoritative source of information on cancer epidemiology and clinical characteristics in the United States. In this retrospective cohort study of fNENs from 2000 to 2018, we used the data from the SEER database (SEER- 17). Patients of fNENs were identified based on the histologic codes from the International Classification of Diseases for Oncology, 3rd Edition (ICD-O-3) (Supplementary Table 1). The inclusion and exclusion criteria are shown in Supplementary Figure 1. Patient characteristics, including age, race, primary site, size of the primary tumor, stage, grade, year of diagnosis, marital status, and survival were obtained from the SEER database. The West China Hospital of Sichuan University institutional review board deemed this study exempt from review and informed consent because the data are freely available.

### Classification of variables

The population was divided into 2 groups by 50 years old firstly because we found a great gap in people younger or older than 50 years old. 10 years were used as a period for detailed analysis and participants younger than 30 or over 80 years old are grouped separately because they are less numerous. We assigned race/ethnicity as Hispanic, non-Hispanic white (NHW), non-Hispanic black (NHB), non-Hispanic Asian or Pacific Islander (NHAPI), and non-Hispanic American Indian/Alaska Native (NHAIAN). The stage was categorized as localized, regional, or distant according to the SEER staging system. The grade was divided into 4 groups: grade (G) 1, well-differentiated; G 2, moderately differentiated; G3, poorly differentiated; G 4, undifferentiated or anaplastic. For the site of the primary tumor, we analyzed breast, female reproductive system (including the uterus, ovary, vulva, and vagina), and other common organs of NENs (appendix, rectum, small intestine, pancreas, lung, stomach, colon, and cecum). Due to the median overall survival (OS) not reaching in some groups, we use 3-year and 5-year survival rates to evaluate the survival as supplementary.

### Nomogram construction and validation

Nomograms, which generate an individual numerical probability of events by integrating diverse prognostic and determinant variables, were used to estimate prognosis [17]. All eligible patients (n = 13,496) were divided into 2:1 training (n = 9,010) and validating (n = 4,505) groups by simple randomization. The factors of interest associated with OS from Multivariable Cox proportional hazards regression models were used to establish the prediction model. The verification of the nomogram is based mainly on the internal (training cohort) and external (validation cohort) discrimination and calibration measurements. The consistency index (C index) was used to evaluate the discriminative ability of the nomogram and the calibration curve was used to compare predicted survival according to the nomogram and actual survival.

### Statistical analysis

Statistical analysis was conducted from December 2022 to March 2023. Descriptive statistics t-tests or χ2 tests were used to compare patients’ basic clinical characteristics.

The age-adjusted incidence was calculated by the SEER*Stat software, version 8.4.0.1. Yearly incidence per 100,000 persons was age-adjusted to the 2000 US standard population. The annual percentage change (APC) was calculated by fitting a simple linear model.

Cox proportional hazards multivariable regression was used to evaluate the association of age, race/ethnicity, Marital status, tumor size, stage, grade, and site with OS by calculating hazard ratios (HRs) and 95% CIs with other factors adjusted. Statistical analyses were conducted with SPSS, version 23 (IBM Corp). All *P*-values were from 2-sided tests, and results were deemed statistically significant at *P* < 0.05.

## RESULTS

### Demographic and clinicopathological characteristics

A total of 39,237 fNEN cases were identified during the study period from the SEER-17 database (Table 1). More patients were diagnosed with fNENs over 50 years old (n=30,108, 76.7%). Among all patients, 63.7% (n=25,010) were white, 14.5% (n=5,682) were black, 13.5% (n=5,293) were Hispanic, and the majority of patients had tumors less than 20 millimeters (n=15,742, 40.1%). In addition, 32.2% (n=12,640) were in G1, 8.1% (n=3,172) were in G2, 7.3% (n=2,845) were in G3, 2.5% (n=979) were in G4, and 50.0% (n=19,601) were unknown. As for the stage, more patients had localized stage (n=16,736, 42.7%), while others had regional (n=5,952, 15.2%) and distant stage (n=7,606, 19.4%). The most common sites of primary cancer were lung (n=9,415, 24.0%), followed by rectum (n=6,958, 17.7%), small intestine (n=5,977, 15.2%), pancreas (n=3,637, 9.3%), appendix (n=3,136, 8.0%), stomach (n=2,746, 7.0%), colon (n=1,244, 3.2%), female reproductive system (n=922, 2.3%), cecum (n=889, 2.3%) and breast (n=252, 0.6%).

**Table 1:** Demographic and clinicopathological characteristics of female neuroendocrine neoplasms (fNENs) patients from 2000 to 2018.

**Variables**	**N**	**%**
**Enrolled patients**	39237	100.0
**Age at initial diagnosis, years**		
< 50	9129	23.3
≥ 50	30108	76.7
**Race**		
Hispanic	5293	13.5
NHW	25010	63.7
NHB	5682	14.5
NHAIAN	259	0.7
NHAPI	2323	5.9
Unknow	670	1.7
**The year of diagnosis**		
2000-2004	6523	16.6
2005-2009	8699	22.2
2010-2014	11566	29.5
2015-2018	12449	31.7
**Marital status**		
Single	6912	17.6
Married	18609	47.4
Divorced/widowed/separated	10154	25.9
Unknown	3562	9.1
**Tumor Size (mm)**		
≤ 20	15742	40.1
21-40	6327	16.1
≥ 41	4626	11.8
Unknown	12542	32.0
**GRADE**		
1	12640	32.2
2	3172	8.1
3	2845	7.3
4	979	2.5
Unknown	19601	50.0
**Disease stage**		
Localized	16736	42.7
Regional	5952	15.2
Distant	7606	19.4
Unstaged	8943	22.8
**Primary tumor sites**		
Appendix	3136	8.0
Rectum	6958	17.7
Small intestine	5977	15.2
Pancreas	3637	9.3
Lung	9415	24.0
Stomach	2746	7.0
Colon	1244	3.2
Reproductive system	922	2.3
Cecum	889	2.3
Breast	252	0.6

NHW indicates Non-Hispanic White; NHB, Non-Hispanic Black; NHAIAN, Non-Hispanic American Indian/Alaska Native; NHAPI, Non-Hispanic Asian or Pacific Islander; mm, millimeter; Grade 1, Well differentiated; Grade 2, Moderately differentiated; Grade 3, Poorly differentiated; Grade 4, Undifferentiated, anaplastic.

### Annual incidence

**Figure 1:**
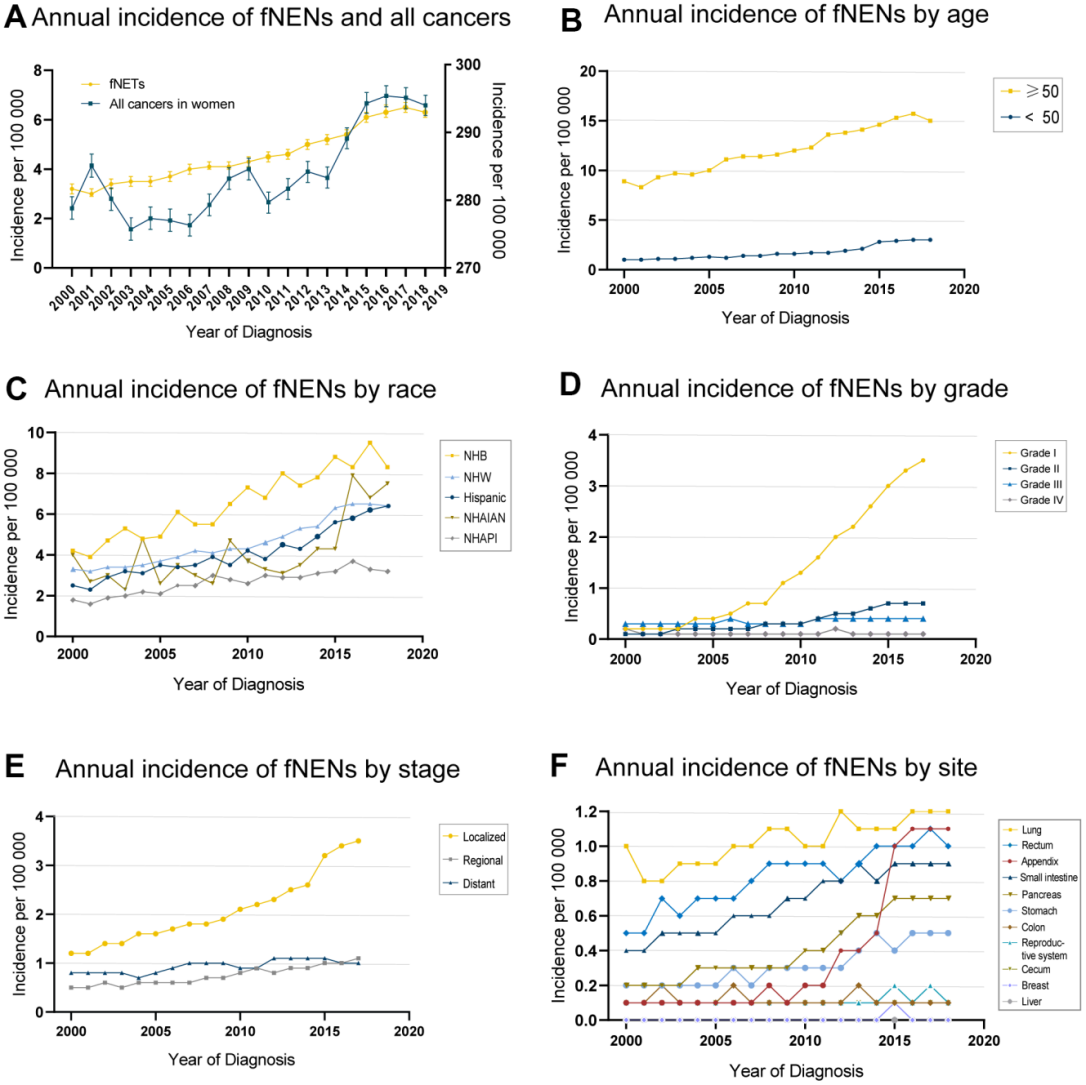
**Morbidity tendency of female neuroendocrine neoplasms (fNENs) patients by age, race, stage, grade, and site.** (**A**) The age-adjusted incidence of women patients in all cancers (right Y) and Neuroendocrine neoplasms (NENs) (left Y) during 2000-2018. (**B**) The fNENs incidence by age. (**C**) The incidence of fNENs in different races. (**D**) The incidence of fNENs by grade. (**E**) Incidence of fNENs by stage. (**F**) The incidence of fNENs by the site.

We used population data from the SEER program to calculate the age-adjusted incidence of fNENs per 100,000 individuals per year with a population of 2000 as the standard population. The age-adjusted incidence of fNENs increased from 3.2 to 6.3 per 100,000 from 2000 to 2018, with an APC of 4.5 (95%CI, 4.1-4.8) (Figure 1A). The increasing incidence of fNENs was found in all age groups, while the majority of fNENs patients (76.7%) were over 50 years old (Figure 1B). This upward trend was also observed in almost all race, grade, and stage groups, with the most noticeable increase in Hispanic (APC, 5.4; 95%CI, 4.7-6.1), Grade 1 (APC, 19.4; 95%CI, 17.6-21.3) and localized fNENs (APC, 6.6; 95%CI, 6.0-7.2) (Figure 1C–1E). With regard to the primary sites, fNENs diagnosed at lung, appendix, rectum, small intestine, pancreas, and stomach increased over time. The most remarkable increases were reported in appendix (APC, 19.3; 95%CI, 16.0-22.7), followed by pancreas (APC, 8.6; 95%CI, 7.7-9.6), stomach (APC, 6.3; 95%CI, 5.4-7.2), small intestine (APC, 4.5; 95%CI, 3.9-5), rectum (APC, 3.7; 95%CI, 2.9-4.5), and lung (APC, 1.8; 95%CI, 1.2-2.4) (Figure 1F).

### Incidence according to site and age

**Figure 2:**
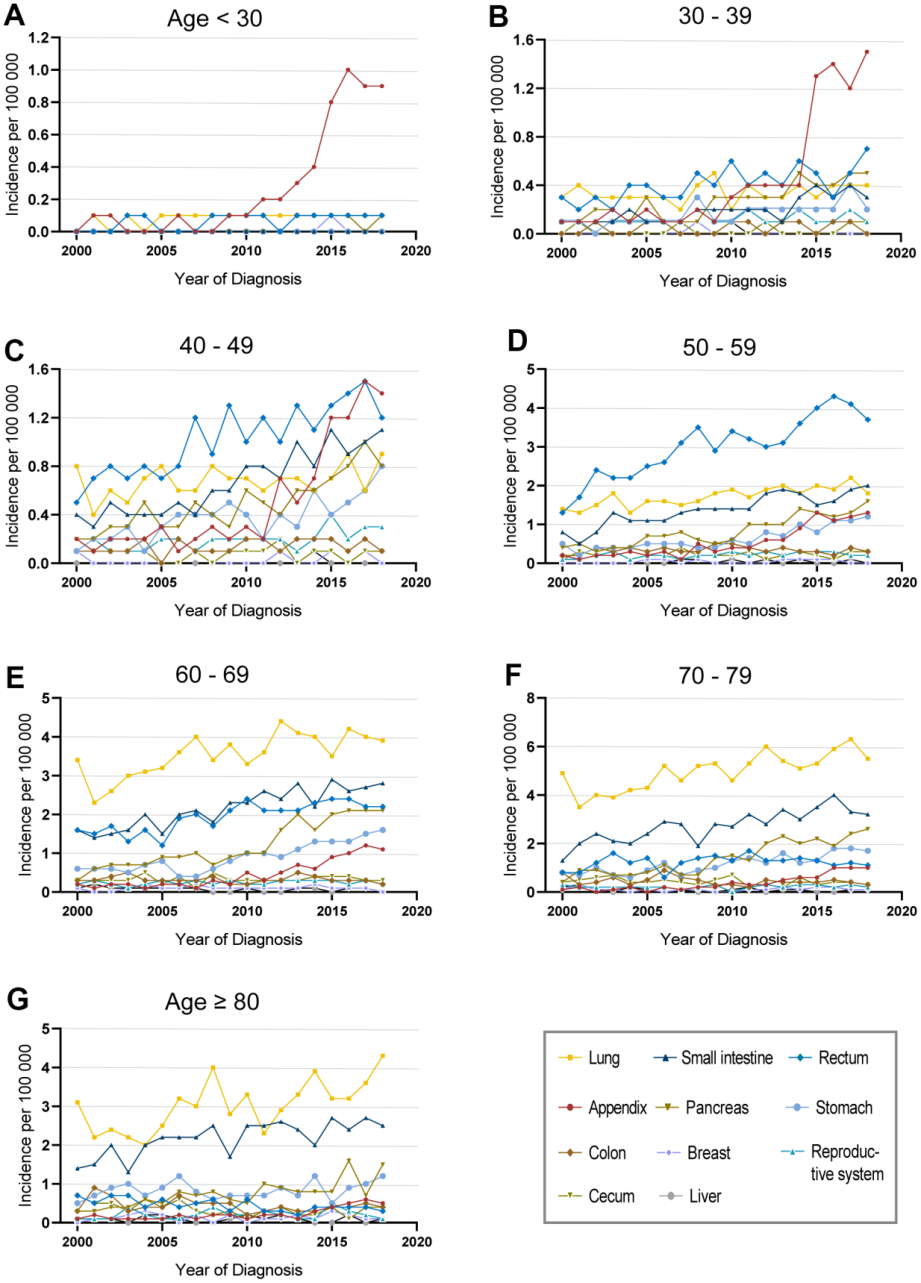
**Incidence of female neuroendocrine neoplasms (fNENs) patients with different sites in various age groups.** The incidence of fNENs by site in patients younger than 30 years old (**A**), between 30-39 years old (**B**), between 40-49 years old (**C**), between 50-59 years old (**D**), between 60-69 years old (**E**), between 70-79 years old (**F**) and over 80 years old (**G**).

Due to the significant disparities of different ages and tumor sites, we programmed an in-depth analysis of the incidence of fNENs patients (Figure 2 and Supplementary Figure 2). The increasing incidence of fNENs was found in almost all age groups (Supplementary Figure 2A). In young fNENs (<40 years old), the overall incidence was low while appendiceal fNENs showed significant growth in the last 10 years (Figure 2A, 2B and Supplementary Figure 2F). Rectum fNENs played a dominant role in patients aged 40-59 years old, and pulmonary fNENs in patients over 60 years old (Figure 2C–2G).

For the age distribution of fNENs patients with common sites, the major population was over 50 years old in pulmonary, pancreatic, and small intestinal fNENs (Supplementary Figure 2B–2D), and 50-59 years old seemed to be the period of high incidence of rectal NENs (Supplementary Figure 2E). Although there were similar increasing trends in all age groups in appendiceal fNENs, the number of young patients was a little bit more (Supplementary Figure 2F).

**Figure 3:**
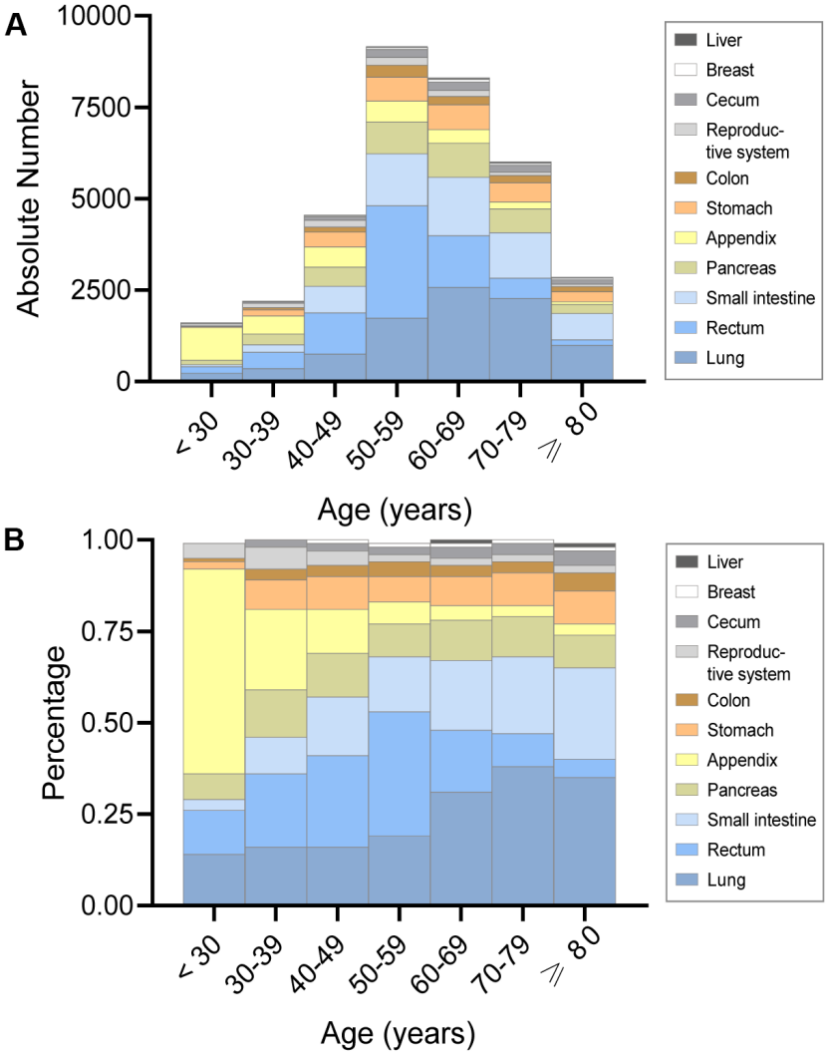
**The absolute number and proportion of incidence in female neuroendocrine neoplasms (fNENs) by site for each age group.** (**A**) The number of fNENs patients with various sites in each age group. (**B**) The proportion of fNENs patients with various sites in each age group.

In terms of overall absolute number and percentage, the lung was the most frequent site of fNENs and was inclined to occur after 50 years old (Figure 3A). Similarly, the number of fNENs located in the stomach, small intestine, rectum, and pancreas had an abrupt growth after 50 years old (Figure 3A). The proportion of fNENs in different sites for each age group is presented in Figure 3B. The results also showed that fNENs of the appendix and female reproductive system have a higher proportion in young patients versus the elderly. Whereas the incidence of pulmonary and intestinal NENs was markedly higher among older than younger women.

### Survival

**Figure 4:**
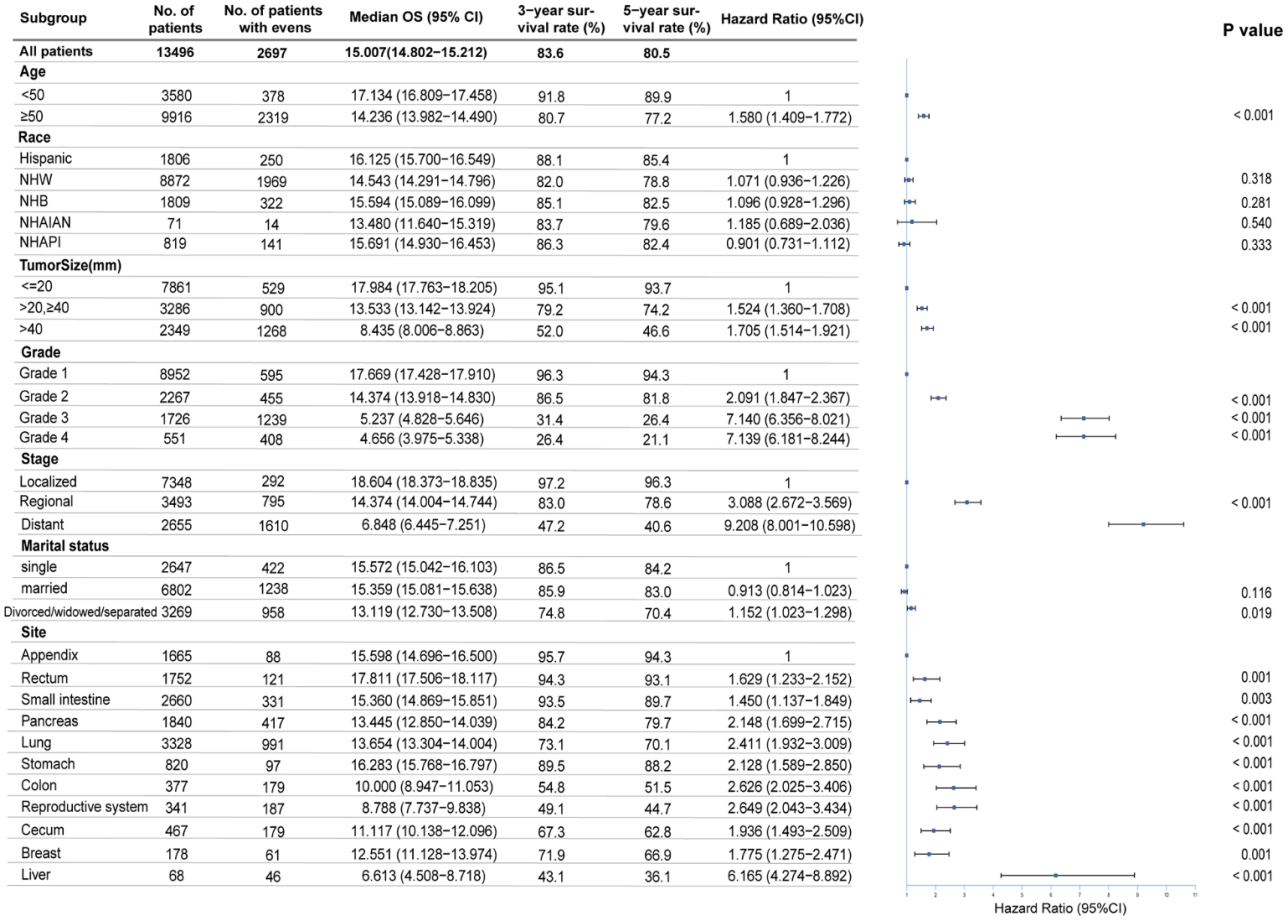
**Multivariable regression analysis for female neuroendocrine neoplasms (fNENs).** OS, overall survival; NHW, Non-Hispanic White; NHB, Non-Hispanic Black; NHAIAN, Non-Hispanic American Indian/Alaska Native; NHAPI, Non-Hispanic Asian or Pacific Islander; mm, millimeter; Grade 1, Well differentiated; Grade 2, Moderately differentiated; Grade 3, Poorly differentiated; Grade 4, Undifferentiated, anaplastic.

The median OS of all fNENs patients was 15.0 years (180.1 months) and 5-year survival was 80.5% (Figure 4). The overall prognosis of young patients was better than that of the elderly and rectal fNENs had the best survival in all site groups (Figure 4). In patients under 50 years old, only pancreatic fNENs reached median OS (17.7 years) and fNENs of the breast had the worst 5-year survival rate (57.6%) (Table 2). While in those older than 50 years old, the worst 5-year survival rate was found in hepatic fNENs. Patients diagnosed with G1 (17.7 years) or G2 (14.4 years) had a significantly better median OS than G3 (5.2 years) or G4 (4.7 years) (Figure 4 and Supplementary Figure 3A). For the different stages, the median OS was 18.6 years in localized,14.4 years in regional, and 6.8 years in distant stages (Figure 4 and Supplementary Figure 3B). There seemed to be no obvious difference in the survival between different races (Figure 4 and Supplementary Figure 3C). The fNENs in rectum (17.8 years), stomach (16.3 years), appendix (15.6 years), and small intestine (15.4 years) had better prognosis among site groups, while fNENs in liver (6.6 years) and female reproductive system (8.8 years) had worse outcomes (Figure 4 and Supplementary Figure 3D).

**Table 2:** Survival analysis of female neuroendocrine neoplasms (fNENs) patients: actuarial survival of fNENs patients diagnosed from 2000 to 2018 by age and primary tumor site.

**Tumor site**	**Age <50**				**Age≥50**		
	**Median survival** **(Years)**	**Survival rate (%)**			**Median survival** **(Years)**	**Survival rate (%)**	
		**3-Year**	**5-Year**			**3-Year**	**5-Year**
All	NR	88.7	86.1		NR	72.3	68.7
best	NR	97.7	97.4		NR	96.7	95.9
Appendix	NR	97.6	95.7		NR	86.9	82.6
Stomach	NR	94.7	95.0		NR	90.0	89.2
Small intestine	NR	97.1	95.0		NR	90.0	85.0
Lung	NR	86.9	85.4		18.5	60.3	57.1
Colon	NR	80.0	76.1		19.5	63.8	61.2
Cecum	NR	79.4	76.7		15.3	67.8	62.6
Pancreas	17.7	80.1	73.5		8.8	66.6	59.6
Breast	NR	62.1	57.6		15.0	68.5	62.9
Reproductive system	NR	65.0	62.1		2.7	49	44.7
Liver	NR	74.8	71.7		1.5	39.8	31.4

Then, we further analyzed the survival outcomes according to age and site (Supplementary Table 2). When age was divided more detailed, rectal fNENs still had the highest survival rate in almost all age groups. The survival of pulmonary fNENs showed a continued and obvious decline after 40 years old. However, the breast fNENs seemed to have the worst survival in young patients (age<40). In addition, the worst survival rate was shown in fNENs of the reproductive system aged 40-49 years old and fNENs of liver in those over 50 years old.

### Multivariable analysis

To identify the independent prognostic risk factors, we performed a multivariable analysis. The Cox proportional hazards regression model revealed that age, tumor size, grade, stage, and primary site were closely related to survival (Figure 4). Compared with the patients younger than 50 years old, the risk of death for the older was increased (HR, 1.58; 95%CI, 1.41-1.77). The prognosis was poorer for fNENs with G3 (HR, 7.14; 95%CI, 6.36-8.02) and G4 (HR, 7.14; 95%CI, 6.18-8.24) than for those with G1 and G2 (HR, 2.091; 95%CI, 1.85-2.37) after adjustment for other covariates. Overall survival was worse for patients with distant NENs (HR, 9.21; 95%CI, 8.00-10.60) than localized NENs. When we used appendiceal NENs as a reference, we observed that fNENs of other sites all had a higher risk of death. Among them, fNENs of small intestine (HR, 2.15, 95%CI, 1.70-2.72), pancreas (HR, 2.41; 95%CI, 1.93-3.01), lung (HR, 2.13, 95%CI, 1.59-2.85), stomach (HR, 2.63, 95%CI, 2.03-3.41), and colon (HR, 2.65; 95%CI, 2.04-3.43) had higher risk of death than appendiceal NENs, in which liver fNENs was the most dangerous (HR, 6.17; 95%CI, 4.27-8.89).

### Nomograms

**Figure 5:**
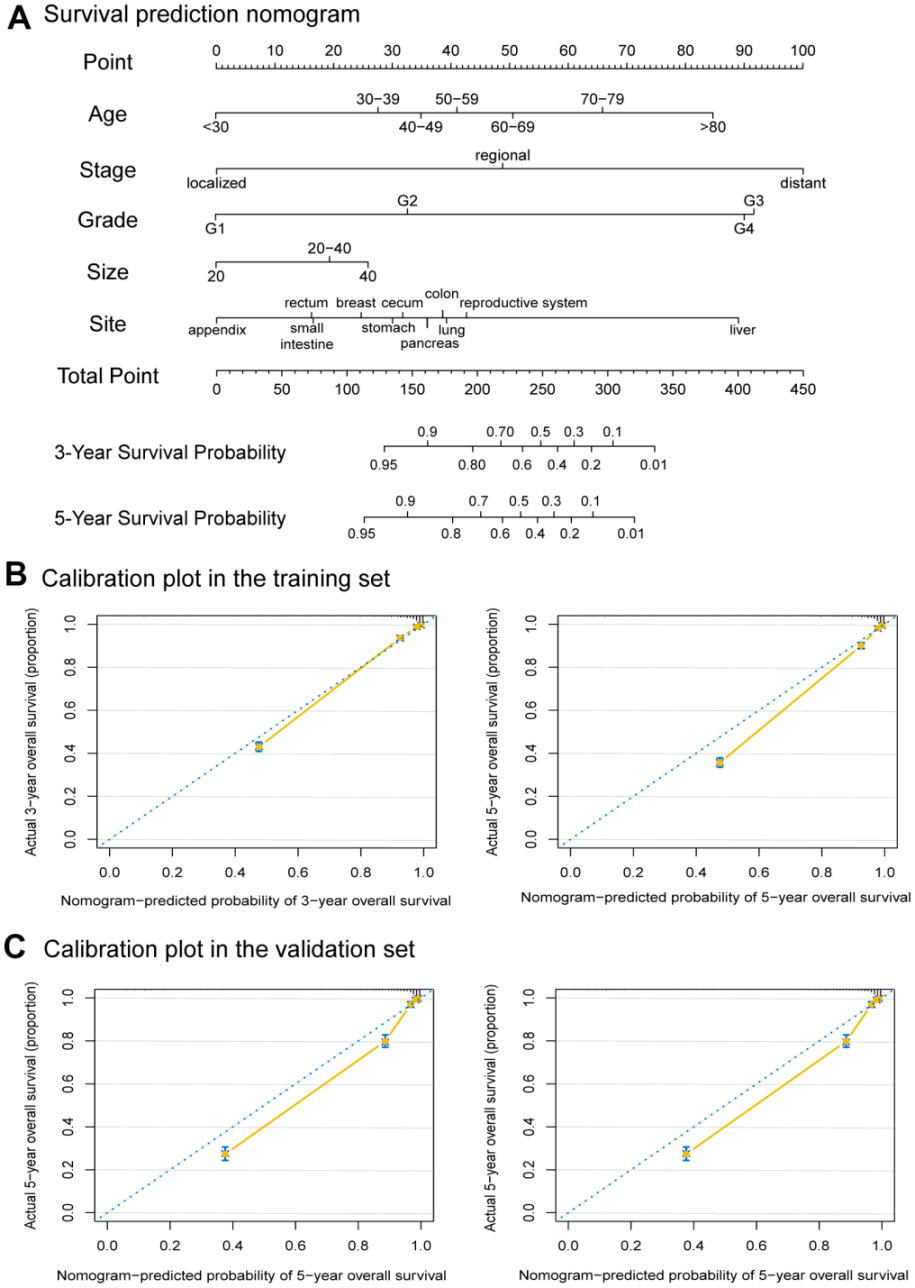
**Nomogram to predict the 3-Year and 5-Year survival probabilities of female neuroendocrine neoplasms (fNENs) patients and the calibration of the nomogram using the training and validation sets.** (**A**) Points for age, disease stage, tumor grade, tumor size, and primary tumor site are obtained by drawing a line upward from the corresponding values to the “Points” line. The sum of the points of these 5 factors is located on the “Total points” line, and a line projected down to the bottom scales determines the probabilities of 3-year and 5-year overall survival (OS). (**B**) Calibration plots of the nomogram for 3-year and 5-year survival probabilities in the training set. (**C**) Calibration plots of the nomogram for 3-year and 5-year survival probabilities in the validation set.

A collection of 13,496 eligible patients from previous cohorts was included in the study according to the model-building requirements (Supplementary Figure 1). Then they were randomly divided into a training set and a validation set by a ratio of 2:1. The training and validation cohorts were comparable in terms of demographic and clinical characteristics (*P* > 0.05) (Supplementary Table 3). According to the results of the Cox proportional hazards regression model, the prediction model contains age, tumor size, grade, stage, and primary site. Consistent with the previous analysis results, the disease stage had the greatest significance, contributing a maximum of 100 points. Grade (91 points), primary tumor site (89 points), age (85 points), and tumor size (26 points) were also individually associated with OS (Figure 5A). Then we internally and externally validated the nomogram, the C indexes for OS prediction of the training cohort (internal validation) and validation cohort (external validation) in the nomogram were 0.906 (95% CI, 0.900-0.912) and 0.901 (95% CI, 0.902-0.918), respectively. Finally, the calibration plots of the nomogram showed consistency between the nomogram-predicted and actual outcomes in the internal (Figure 5B) and external (Figure 5C) validation.

## DISCUSSION

Previous studies have reported the increased incidence of NENs worldwide, in which approximately 50% were female patients [14, 15]. Gender differences in the incidence, diagnosis, and prognosis of NENs have been reported, while there is still a lack of overall epidemiological description and prognostic risk assessment for fNENs patients [14, 18, 19]. Our analysis for fNENs discovered that fNENs of appendix, rectum, and lung had the highest incidence in the young (<40), middle-aged (40-59), and elderly (≥60), respectively. Additionally, fNENs of the breast, reproductive system, and liver had the worst survival in patients younger than 50, between 50 to 59, and those older than 60 years old, respectively. At the same time, the predictive models incorporating detailed age and site also showed superior predictive power. Our results demonstrated that gender differences are also worthy of attention in fNEN. The distinct tendency in various age groups with different tumor sites confirmed the importance and necessity of implementing early screening, patient stratification, and precision medicine for specific populations with different ages and sites.

Consistent with previous reports, the age-adjusted annual incidence of fNENs has gradually risen over the past decade, in which Grade 1 and localized fNENs rose markedly [14, 15]. This is possibly attributed to the detection of asymptomatic early-stage disease after the extensive use of endoscopy and computed tomography [20]. Notably, the morbidity of different sites presented obvious discriminations. Developments in imaging examinations make fNENs at lung, rectum, small intestine, pancreas, and stomach easier to be diagnosed and therefore benefit a lot. Although fNENs of the lung, rectum and intestine occupy the majority, the appendiceal fNENs showed an unexpectedly tremendous increase (11-fold). Interestingly, the phenomenon was observed in recent years, partly attributed to a better understanding of the pathophysiologic presentation of NENs, improved classification system, as well as availability of advanced imaging modalities [20–22]. Another reason may be that pelvic surgery increased the probability of finding appendiceal fNEN [23]. Furthermore, we noticed that the population of patients diagnosed after 50 years old was larger than the younger and approximately accounted for four-fifths of fNENs. At the same time, the number of patients aged 50-59 was twice as many as patients aged 40-49 years old, indicating that menopause (about 50 years old) significantly affects the incidence.

Previous studies demonstrate an age-dependent pattern for nearly all pancreatic cancer risk factors [24]. A previous epidemiological survey of NENs also showed that a more significant growth of incidence was found in the older, suggesting that age has a huge impact on the morbidity of NENs [15]. Thus, considering the enormous effects of different ages and sites, we evaluated the incidence patterns according to site and detailed age groups. The frequency distribution of incidence by site and age corroborated our hypotheses that the occurrence of fNENs in different sites is partially age-dependent. Different from previous studies, appendiceal NENs were more diagnosed in women younger than 40 and the number decreased as people advanced in age [25]. The pelvic surgery made appendiceal NENs more frequent and the age of diagnosis is getting younger and younger [23]. Thus, appendiceal NENs in young women deserve more attention, corresponding screening and treatment strategies might be made for them [26]. Further analysis revealed that the upstart rectal fNENs play a major role in patients between 40-59 and pulmonary fNENs in patients over 60 years old, suggesting that appropriate screening time and site should be considered for different populations. The incidence of fNEN in the lung, pancreas, rectum, and small intestine has doubled after the age of 50, and the proportion of fNENs younger than 40 years old is the highest among appendiceal fNEN, which coincidentally corresponds to the weakest (menopause) and most vigorous (gestation period) period of female hormones. This age-dependent incidence pattern makes us pay attention to the role of female hormones in it. In reported small intestinal NENs, the increased risk of mesenteric metastasis in women around menopause also presented the role of hormones in fNENs [27]. In a word, age differences in fNENs may be attributed to the effects of different hormone levels on different organs. However, more investigations are required to explore whether and how hormone secretion status influences the fNENs in different sites.

The 5-years survival estimate for fNENs patients was 15.0 years, higher than that of reported overall NENs patients [14, 15]. Given the mismatching of diagnostic time, we cannot conclusively state that women have better prognoses. Different from the results of the whole NENs population, we found that fNENs in the rectum had the best median OS among site groups, a little longer than that in appendix [14, 15]. While the worst outcomes were shown in hepatic NENs. For women patients, NENs of breast and reproductive system deserve more attention, mainly because they are too rare to be checked timely. Similarly, we analyzed the relationship between survival, age, and site, finding that breast, reproductive system, and liver fNENs had the lowest 5-year survival rate in patients younger than 40, between 40-49, and over 50 years old, respectively. Therefore, clinicians should raise awareness of rare fNENs and carry out timely and effective treatment.

As reported, age, disease stage, grade, size, and primary tumor site were important predictors of survival outcomes [14, 15]. Our analyses did not observe a statistically significant difference in survival period among patients of different races and years of diagnosis, indicating better representativeness and applicability. Consistently, the disease stage had the greatest significance, contributing a maximum of 100 points. At the same time, age (85 points) and site (89 points) also had a great influence on survival, which was not observed in another nomogram whose age groups were simply divided [28]. More detailed division and higher weight of age made our nomogram model have higher predictive power [28, 29]. The need for precise analysis of clinical characteristics makes the use of more detailed age groups in large data-based studies a future research trend [24, 30]. As our nomogram’s good performance and high compatibility were confirmed, it will provide a preliminary assessment for patient stratification and precision therapy, which would be more helpful for practicing clinicians. However, the treatments were not contained in the prognostic model because of the limitation of the database. The treatments for NENs include somatostatin analogue therapy, molecularly targeted agents (for example, everolimus and sunitinib), peptide receptor radionuclide therapy with Lutetium-177 DOTA-TATE (177Lu PRRT), chemotherapy, and surgery, which were not as detailed as we wished in it [5]. Despite all this, our nomograms still show a high accuracy, which could provide a preliminary prognostic evaluation for fNENs patients.

There were several limitations in our study. First, the use of the database had natural lag and potential bias, which may cause some discrepancies from the true incidence of NENs. Due to the limitations of accessible information, we took more consideration of baseline characteristics and lacked more detailed patient-level data beyond basic demographics and tumor characteristics. Then, we did not perform external validation with an independent cohort to further confirm the generalizability. However, the SEER database initially provided a comprehensive epidemiologic picture of fNENs, which helped us to get first-hand data for guiding clinical practice. The SEER database has its own stage and grade system, which differs somewhat from the latest proposed classification [31, 32]. And we look forward to the update of the SEER system that will provide more clinical information in the future.

## CONCLUSIONS

The incidence of fNENs has continued to rise from 2000 to 2018. The incidence and survival pattern presented obvious differences in various age and site groups, emphasizing the necessity of making specific screening strategies for patients of different ages. The poor prognosis of fNENs in uncommon sites, such as the breast, reproductive system, and liver also deserved more attention. The nomogram based on this data had been proven to be accurate and reliable, indicating the application for preliminary patient evaluation. However, we need to collect more clinical information further to improve the accuracy of the model in the future.

## Supplementary Material

Supplementary Figures

Supplementary Tables

## References

[1] Hofland J, Kaltsas G, de Herder WW. Advances in the Diagnosis and Management of Well-Differentiated Neuroendocrine Neoplasms. Endocr Rev. 2020; 41:371–403. 10.1210/endrev/bnz004 31555796 PMC7080342

[2] Sheikhbahaei S, Sadaghiani MS, Rowe SP, Solnes LB. Neuroendocrine Tumor Theranostics: An Update and Emerging Applications in Clinical Practice. AJR Am J Roentgenol. 2021; 217:495–506. 10.2214/AJR.20.23349 34076455

[3] La Rosa S, Uccella S. Classification of neuroendocrine neoplasms: lights and shadows. Rev Endocr Metab Disord. 2021; 22:527–38. 10.1007/s11154-020-09612-2 33169199 PMC8346451

[4] van der Graaf WTA, Tesselaar ME, McVeigh TP, Oyen WJG, Fröhling S. Biology-guided precision medicine in rare cancers: Lessons from sarcomas and neuroendocrine tumours. Semin Cancer Biol. 2022; 84:228–41. 10.1016/j.semcancer.2022.05.011 35643220

[5] Caplin ME, Ratnayake GM. Diagnostic and therapeutic advances in neuroendocrine tumours. Nat Rev Endocrinol. 2021; 17:81–2. 10.1038/s41574-020-00458-x 33335329

[6] Luo G, Javed A, Strosberg JR, Jin K, Zhang Y, Liu C, Xu J, Soares K, Weiss MJ, Zheng L, Wolfgang CL, Cives M, Wong J, et al. Modified Staging Classification for Pancreatic Neuroendocrine Tumors on the Basis of the American Joint Committee on Cancer and European Neuroendocrine Tumor Society Systems. J Clin Oncol. 2017; 35:274–80. 10.1200/JCO.2016.67.8193 27646952

[7] Walter T, Lievre A, Coriat R, Malka D, Elhajbi F, Di Fiore F, Hentic O, Smith D, Hautefeuille V, Roquin G, Perrier M, Dahan L, Granger V, et al. Bevacizumab plus FOLFIRI after failure of platinum-etoposide first-line chemotherapy in patients with advanced neuroendocrine carcinoma (PRODIGE 41-BEVANEC): a randomised, multicentre, non-comparative, open-label, phase 2 trial. Lancet Oncol. 2023; 24:297–306. 10.1016/S1470-2045(23)00001-3 36739879

[8] LeClair K, Bell KJL, Furuya-Kanamori L, Doi SA, Francis DO, Davies L. Evaluation of Gender Inequity in Thyroid Cancer Diagnosis: Differences by Sex in US Thyroid Cancer Incidence Compared With a Meta-analysis of Subclinical Thyroid Cancer Rates at Autopsy. JAMA Intern Med. 2021; 181:1351–8. 10.1001/jamainternmed.2021.4804 34459841 PMC8406211

[9] Smith AJ, Lambert PC, Rutherford MJ. Understanding the impact of sex and stage differences on melanoma cancer patient survival: a SEER-based study. Br J Cancer. 2021; 124:671–7. 10.1038/s41416-020-01144-5 33144697 PMC7851379

[10] Goodman WA, Erkkila IP, Pizarro TT. Sex matters: impact on pathogenesis, presentation and treatment of inflammatory bowel disease. Nat Rev Gastroenterol Hepatol. 2020; 17:740–54. 10.1038/s41575-020-0354-0 32901108 PMC7750031

[11] Man JJ, Beckman JA, Jaffe IZ. Sex as a Biological Variable in Atherosclerosis. Circ Res. 2020; 126:1297–319. 10.1161/CIRCRESAHA.120.315930 32324497 PMC7185045

[12] Shah S, Gosain R, Groman A, Gosain R, Dasari A, Halfdanarson TR, Mukherjee S. Incidence and Survival Outcomes in Patients with Lung Neuroendocrine Neoplasms in the United States. Cancers (Basel). 2021; 13:1753. 10.3390/cancers13081753 33916960 PMC8067543

[13] Patel N, Benipal B. Incidence of Neuroendocrine Tumors in the United States from 2001-2015: A United States Cancer Statistics Analysis of 50 States. Cureus. 2019; 11:e4322. 10.7759/cureus.4322 31183301 PMC6538402

[14] Yao JC, Hassan M, Phan A, Dagohoy C, Leary C, Mares JE, Abdalla EK, Fleming JB, Vauthey JN, Rashid A, Evans DB. One hundred years after “carcinoid”: epidemiology of and prognostic factors for neuroendocrine tumors in 35,825 cases in the United States. J Clin Oncol. 2008; 26:3063–72. 10.1200/JCO.2007.15.4377 18565894

[15] Dasari A, Shen C, Halperin D, Zhao B, Zhou S, Xu Y, Shih T, Yao JC. Trends in the Incidence, Prevalence, and Survival Outcomes in Patients With Neuroendocrine Tumors in the United States. JAMA Oncol. 2017; 3:1335–42. 10.1001/jamaoncol.2017.0589 28448665 PMC5824320

[16] Gupta N, Reddy K, Gnanasekaran P, Zhai Y, Chakraborty S, Pappu HR. Functional characterization of a new ORF βV1 encoded by radish leaf curl betasatellite. Front Plant Sci. 2022; 13:972386. 10.3389/fpls.2022.972386 36212370 PMC9546537

[17] Balachandran VP, Gonen M, Smith JJ, DeMatteo RP. Nomograms in oncology: more than meets the eye. Lancet Oncol. 2015; 16:e173–80. 10.1016/S1470-2045(14)71116-7 25846097 PMC4465353

[18] Tam M, Luu M, Barker CA, Gharavi NM, Hamid O, Shiao SL, Nguyen AT, Lu DJ, Ho AS, Zumsteg ZS. Improved survival in women versus men with merkel cell carcinoma. J Am Acad Dermatol. 2021; 84:321–9. 10.1016/j.jaad.2020.02.034 32423829

[19] Gaur P, Leary C, Yao JC. Thymic neuroendocrine tumors: a SEER database analysis of 160 patients. Ann Surg. 2010; 251:1117–21. 10.1097/SLA.0b013e3181dd4ec4 20485130

[20] Ruf J, von Wedel F, Furth C, Denecke T, Stelter L, Steffen IG, Schütte K, Arend J, Ulrich G, Klose S, Bornschein J, Apostolova I, Amthauer H. Significance of a Single-Time-Point Somatostatin Receptor SPECT/Multiphase CT Protocol in the Diagnostic Work-up of Gastroenteropancreatic Neuroendocrine Neoplasms. J Nucl Med. 2016; 57:180–5. 10.2967/jnumed.115.161117 26609177

[21] Mohamed A, Wu S, Hamid M, Mahipal A, Cjakrabarti S, Bajor D, Selfridge JE, Asa SL. Management of Appendix Neuroendocrine Neoplasms: Insights on the Current Guidelines. Cancers (Basel). 2022; 15:295. 10.3390/cancers15010295 36612291 PMC9818268

[22] Wang H, Cheng Y, Zhang J, Zang J, Li H, Liu Q, Wang J, Jacobson O, Li F, Zhu Z, Chen X. Response to Single Low-dose 177Lu-DOTA-EB-TATE Treatment in Patients with Advanced Neuroendocrine Neoplasm: A Prospective Pilot Study. Theranostics. 2018; 8:3308–16. 10.7150/thno.25919 29930731 PMC6010978

[23] Teixeira FJR Jr, Couto Netto SDD, Akaishi EH, Utiyama EM, Menegozzo CAM, Rocha MC. Acute appendicitis, inflammatory appendiceal mass and the risk of a hidden malignant tumor: a systematic review of the literature. World J Emerg Surg. 2017; 12:12. 10.1186/s13017-017-0122-9 28286544 PMC5343298

[24] Yuan C, Kim J, Wang QL, Lee AA, Babic A, Amundadottir LT, Klein AP, Li D, McCullough ML, Petersen GM, Risch HA, Stolzenberg-Solomon RZ, Perez K, et al, and PanScan/PanC4 I-III Consortium. The age-dependent association of risk factors with pancreatic cancer. Ann Oncol. 2022; 33:693–701. 10.1016/j.annonc.2022.03.276 35398288 PMC9233063

[25] Vinagre J, Pinheiro J, Martinho O, Reis RM, Preto J, Soares P, Lopes JM. A 30-Year Long-Term Experience in Appendix Neuroendocrine Neoplasms-Granting a Positive Outcome. Cancers (Basel). 2020; 12:1357. 10.3390/cancers12061357 32466539 PMC7353034

[26] Nesti C, Bräutigam K, Benavent M, Bernal L, Boharoon H, Botling J, Bouroumeau A, Brcic I, Brunner M, Cadiot G, Camara M, Christ E, Clerici T, et al. Hemicolectomy versus appendectomy for patients with appendiceal neuroendocrine tumours 1-2 cm in size: a retrospective, Europe-wide, pooled cohort study. Lancet Oncol. 2023; 24:187–94. 10.1016/S1470-2045(22)00750-1 36640790

[27] Blažević A, Iyer AM, van Velthuysen MF, Hofland J, Oudijk L, de Herder WW, Hofland LJ, Feelders RA. Sexual Dimorphism in Small-intestinal Neuroendocrine Tumors: Lower Prevalence of Mesenteric Disease in Premenopausal Women. J Clin Endocrinol Metab. 2022; 107:e1969–75. 10.1210/clinem/dgac001 34999838 PMC9016466

[28] Fang C, Wang W, Feng X, Sun J, Zhang Y, Zeng Y, Wang J, Chen H, Cai M, Lin J, Chen M, Chen Y, Li Y, et al. Nomogram individually predicts the overall survival of patients with gastroenteropancreatic neuroendocrine neoplasms. Br J Cancer. 2017; 117:1544–50. 10.1038/bjc.2017.315 28949958 PMC5680463

[29] Xu Z, Wang L, Dai S, Chen M, Li F, Sun J, Luo F. Epidemiologic Trends of and Factors Associated With Overall Survival for Patients With Gastroenteropancreatic Neuroendocrine Tumors in the United States. JAMA Netw Open. 2021; 4:e2124750. 10.1001/jamanetworkopen.2021.24750 34554237 PMC8461504

[30] Paulson KG, Gupta D, Kim TS, Veatch JR, Byrd DR, Bhatia S, Wojcik K, Chapuis AG, Thompson JA, Madeleine MM, Gardner JM. Age-Specific Incidence of Melanoma in the United States. JAMA Dermatol. 2020; 156:57–64. 10.1001/jamadermatol.2019.3353 31721989 PMC6865303

[31] Rindi G, Mete O, Uccella S, Basturk O, La Rosa S, Brosens LAA, Ezzat S, de Herder WW, Klimstra DS, Papotti M, Asa SL. Overview of the 2022 WHO Classification of Neuroendocrine Neoplasms. Endocr Pathol. 2022; 33:115–54. 10.1007/s12022-022-09708-2 35294740

[32] Rindi G, Klimstra DS, Abedi-Ardekani B, Asa SL, Bosman FT, Brambilla E, Busam KJ, de Krijger RR, Dietel M, El-Naggar AK, Fernandez-Cuesta L, Klöppel G, McCluggage WG, et al. A common classification framework for neuroendocrine neoplasms: an International Agency for Research on Cancer (IARC) and World Health Organization (WHO) expert consensus proposal. Mod Pathol. 2018; 31:1770–86. 10.1038/s41379-018-0110-y 30140036 PMC6265262

