# Supplementary material for: The crucial role of age and site in incidence and prognosis of female neuroendocrine neoplasms in the United States: a population-based study from 2000 to 2018
Source: Aging (Albany NY). 2024 Mar 1;16(5):4204–23. doi: 10.18632/aging.205573 (PMC10968707; doi:10.18632/aging.205573)
Supplement: Supplementary Figures [file aging-16-205573-s001.pdf]

SUPPLEMENTARY FIGURES

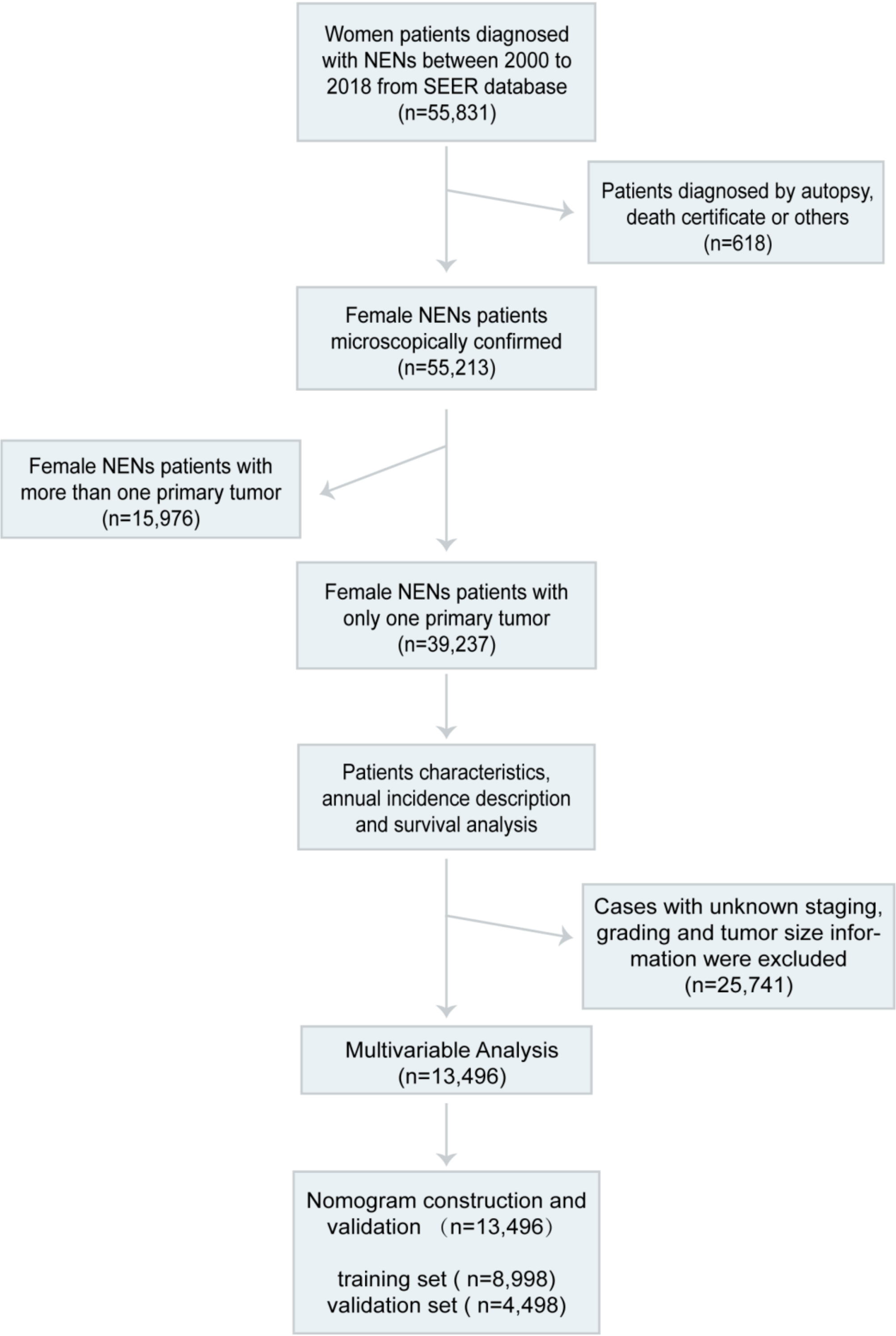

Supplementary Figure 1. Flowchart of patient selection and study design.

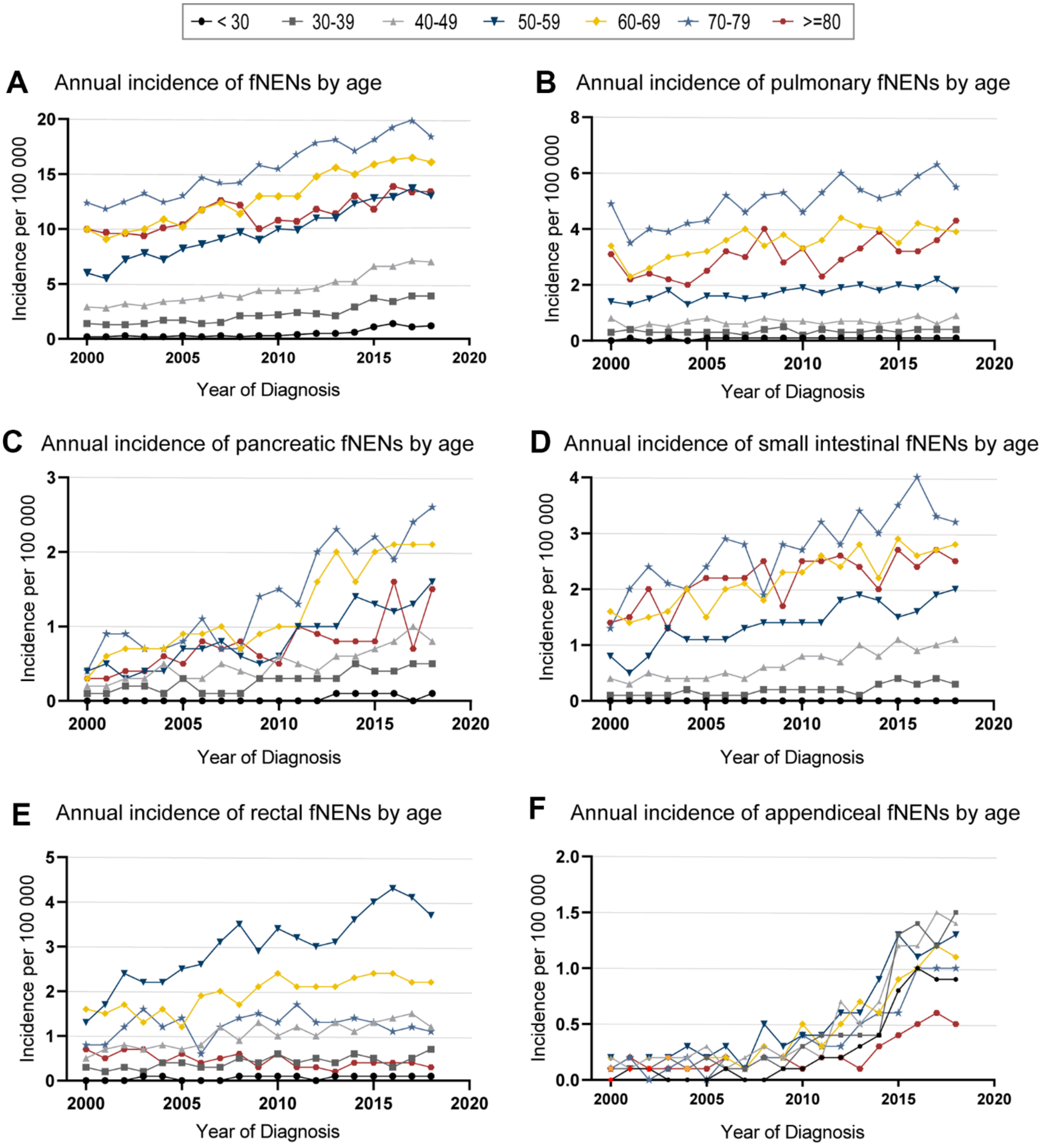

Supplementary Figure 2. Incidence in total and common sites of female neuroendocrine neoplasms (fNENs) by detailed age. The incidence on detailed age in all fNENs (**A**), pulmonary fNENs (**B**), pancreatic fNENs (**C**), intestinal fNENs (**D**), rectal fNENs (**E**), and appendiceal fNENs (**F**).

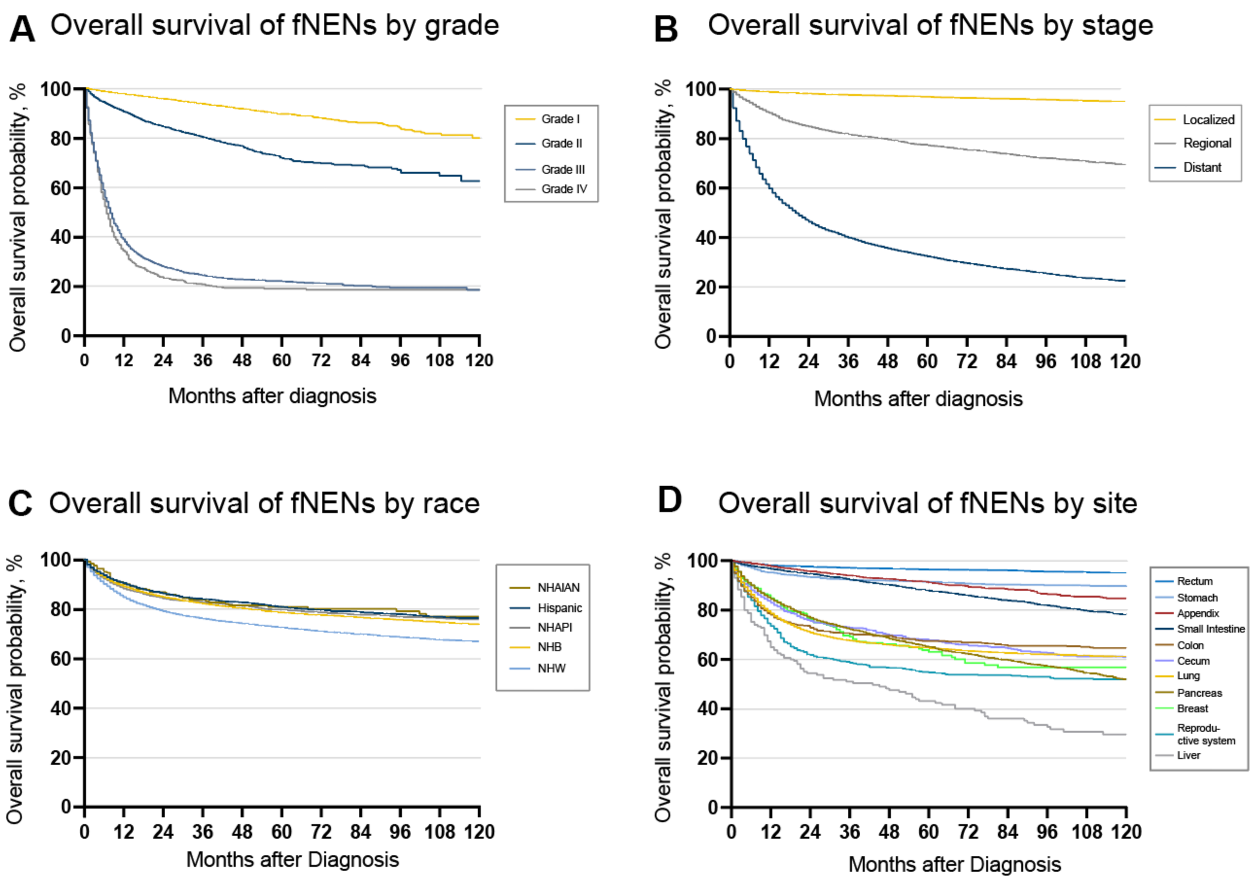

Supplementary Figure 3. The survival curve of fNENs by grade, stage, race, and sites. The survival curve of fNENs by grade (**A**), stage (**B**), race (**C**), and primary site (**D**).
