# Supplementary material for: The crucial role of age and site in incidence and prognosis of female neuroendocrine neoplasms in the United States: a population-based study from 2000 to 2018
Source: Aging (Albany NY). 2024 Mar 1;16(5):4204–23. doi: 10.18632/aging.205573 (PMC10968707; doi:10.18632/aging.205573)
Supplement: Supplementary Tables [file aging-16-205573-s002.pdf]

SUPPLEMENTARY TABLES

Supplementary Table 1. Histologic codes from the international classification of disease for oncology, 3rd edition (ICD-O-3) used to identify neuroendocrine neoplasms (NENs).

NEN histology	ICD code
Pancreatic endocrine tumor	8150/3
Insulinoma	8151/3
Glucagonoma	8152/3
Gastrinoma	8153/3
Mixed pancreatic endocrine and exocrine tumor	8154/3
VIPoma	8155/3
Somatostatinoma	8156/3
Carcinoid tumor	8240/3
Enterochromaffin cell carcinoid	8241/3
Enterochromaffin-like cell tumors	8242/3
Goblet cell carcinoid	8243/3
Mixed adenoneuroendocrine carcinoma	8244/3
Adenocarcinoid tumor	8245/3
Neuroendocrine carcinoma	8246/3
Atypical carcinoid tumor	8249/3

Supplementary Table 2. The median OS and survival rate of female neuroendocrine neoplasms (fNENs) patients by detailed age and primary tumor site.

Variables	Total	Lung	Rectum	Small intestine	Pancreas	Appendix	Stomach	Colon	Reproductive system	Cecum	Breast	Liver
**Total**	39237	8873	6958	5977	3637	3136	2746	1103	922	889	252	191
Median OS (Month)	180.1	163.8	213.7	184.3	161.3	187.2	195.4	120.0	105.3	133.4	150.6	79.4
**<30**	1708	221	185	56	117	897	31	17	59	12	3	7
Median OS		NR	NR	NR	NR	NR	NR	NR	NR	NR	12.0	NR
3-Year SR		95.7	98.8	98.2	83.2	99.8	93.4	82.4	70.4	83.3	33.3	71.4
5-Year SR		95.7	98.8	98.2	77.7	99.4	93.4	82.4	70.4	83.3	33.3	71.4
**30-39**	2413	350	446	211	289	491	175	56	123	33	12	14
Median OS		NR	NR	NR	NR	NR	NR	NR	NR	NR	41.0	NR
3-Year SR		95.4	98.3	98.5	83.5	98.7	94.3	85.1	66.2	87.8	58.3	63.5
5-Year SR		94.7	97.7	97.8	76.0	97.0	92.5	80.4	62.2	87.8	48.6	63.5
**40-49**	5008	750	1122	733	527	551	405	129	193	90	36	15
Median OS		NR	NR	NR	212.0	NR	NR	NR	NR	NR	NR	143.0
3-Year SR		80.3	97.3	96.6	77.7	93.3	94.9	77.4	62.7	76.0	66.0	86.7
5-Year SR		78.0	97.0	93.9	71.2	89.4	94.6	73.3	59.6	72.1	62.8	79.4
**50-59**	9970	1728	3079	1417	870	569	658	330	210	224	46	38
Median OS		NR	NR	NR	148.0	NR	NR	NR	NR	NR	NR	37.0
3-Year SR		70.5	98.6	95.3	73.3	88.2	93.8	83.1	59.6	79.0	69.3	51.6
5-Year SR		67.8	98.3	92.4	65.9	83.8	93.6	81.9	56.6	75.0	60.8	36.6
**60-69**	9421	2571	1416	1599	931	368	682	226	171	229	66	44
Median OS		NR	NR	NR	114.0	NR	NR	NR	48.0	167.0	NR	23.0
3-Year SR		62.3	96.0	92.3	69.6	89.0	93.2	67.6	52.9	71.6	76.6	45.4
5-Year SR		59.5	95.4	87.6	62.4	84.0	92.2	65.7	48.1	67.2	69.6	42.9
**70-79**	7102	2263	557	1246	657	183	525	200	97	186	48	45
Median OS		69.0	NR	NR	80.0	NR	NR	24.0	10.0	62.0	180.0	21.0
3-Year SR		54.6	91.9	86.4	60.6	81.6	87.8	45.5	32.4	56.5	65.0	38.7
5-Year SR		51.0	89.5	80.8	54.8	78.5	86.7	40.0	28.0	49.4	58.2	23.2
**≥ 80**	3615	990	153	715	246	77	270	145	69	115	41	28
Median OS		36.0	NR	165.0	28.0	NR	NR	9.0	7.0	44.0	71.0	7.0
3-Year SR		50.0	80.7	79.5	46.2	80.5	76.9	37.9	27.8	54.1	57.8	15.8
5-Year SR		45.2	75.7	69.5	38.7	80.5	75.4	34.7	20.6	44.9	57.8	15.8

NR, means not reached; OS, overall survival; SR, survival rate (%).

Supplementary Table 3. Baseline clinicopathological characteristics of fNENs patients in the training and validation cohorts.

Variables	All patients (n=13496) N (%)	Training set (n=8998) N (%)	Validation set (n=4498) N (%)	P-value
**Age**				P>0.05
<50	3580(26.5)	2355(26.17)	1225(27.23)	
≥50	9916(73.5)	6643(73.83)	3273(72.77)	
**Race**				P>0.05
Hispanic	1806(13.38)	1223(13.59)	583(12.96)	
NHW	8872(65.74)	5900(65.57)	2972(66.07)	
NHB	1809(13.40)	1217(13.53)	592(13.16)	
NHAIAN	71(0.53)	46(0.51)	25(0.56)	
NHAPI	819(6.07)	535(5.95)	284(6.31)	
Unknow	119(0.88)	77(0.86)	42(0.93)	
**The year of diagnosis**				P>0.05
2000-2004	963(7.14)	647(7.19)	316(7.03)	
2005-2009	2022(14.98)	1341(14.90)	681(15.14)	
2010-2014	5266(39.02)	3501(38.91)	1765(39.24)	
2015-2018	5245(38.86)	3509(39.00)	1736(38.59)	
**Marital status**				P>0.05
Single	2647(19.61)	1765(19.62)	882(19.61)	
Married	6802(50.40)	4584(50.94)	2218(49.31)	
Divorced/widowed/separated	3269(24.22)	2139(23.77)	1130(25.12)	
Unknown	778(5.76)	510(5.67)	268(5.96)	
**Tumor Size (mm)**				P>0.05
≤ 20	7861(58.25)	5186(57.64)	2675(59.47)	
21-40	3286(24.35)	2232(24.81)	1054(23.43)	
≥ 41	2349(17.41)	1580(17.56)	769(17.10)	
**GRADE**				P>0.05
1	8952(66.33)	5934(65.95)	3018(67.10)	
2	2267(16.80)	1545(17.17)	722(16.05)	
3	1726(12.79)	1144(12.71)	582(12.94)	
4	551(4.08)	375(4.17)	176(3.91)	
**Disease stage**				P>0.05
Localized	7348(54.45)	4890(54.35)	2458(54.65)	
Regional	3493(25.88)	2386(26.52)	1107(24.61)	
Distant	2655(19.67)	1722(19.14)	933(20.74)	
**Primary tumor sites**				P>0.05
Appendix	1665(12.34)	1103(12.26)	562(12.49)	
Rectum	1752(12.98)	1163(12.93)	589(13.09)	
Small intestine	2660(19.71)	1767(19.64)	893(19.85)	
Pancreas	1840(13.63)	1217(13.53)	623(13.85)	
Lung	3328(24.66)	2234(24.83)	1094(24.32)	
Stomach	820(6.08)	542(6.02)	278(6.18)	
Colon	377(2.79)	241(2.68)	136(3.02)	
Cecum	467(3.46)	321(3.57)	146(3.25)	
Reproductive system	341(2.52)	237(2.63)	104(2.31)	
Breast	178(1.32)	126(1.40)	52(1.16)	
liver	68(0.50)	47(0.52)	21(0.47)	

NHW indicates Non-Hispanic White; NHB, Non-Hispanic Black; NHAIAN, Non-Hispanic American Indian/Alaska Native; NHAPI, Non-Hispanic Asian or Pacific Islander; mm, millimeter; Grade 1, Well differentiated; Grade 2, Moderately differentiated; Grade 3, Poorly differentiated; Grade 4, Undifferentiated, anaplastic.
